# Unusual Presentation of Post-coital Spontaneous Coronary Artery Dissection

**DOI:** 10.7759/cureus.17460

**Published:** 2021-08-26

**Authors:** Iyad Farouji, Omar Al-Radideh, Hossam Abed, Theodore R DaCosta, Arwa Battah, Amaar S Ahmad, Irvin Goldfarb, Addi Suleiman

**Affiliations:** 1 Internal Medicine, Saint Michael's Medical Center, Newark, USA; 2 Medical Education, Saint Michael's Medical Center, Newark, USA; 3 Cardiology, Saint Michael's Medical Center, Newark, USA

**Keywords:** atypical spontaneous coronary artery dissection, emergency, hypertension, heart, postcoital

## Abstract

Spontaneous coronary artery dissection (SCAD) is a grave medical condition that is defined as a separation of the coronary artery wall layer. This presentation is rare in males and can be triggered by cardio-circulatory stress, such as exercise and emotional stress. Sexual intercourse is considered potent cardiovascular stress that can be strenuous and cause rapid and significant changes in the heart rate and blood pressure which can predispose SCAD. Herein, we are reporting a very rare case of a 41-year-old male gentleman who presented with SCAD after vigorous sexual intercourse. We are reporting this case to encourage physicians to educate their patients on the topic.

## Introduction

Spontaneous coronary artery dissection (SCAD) is a serious medical condition increasing in diagnosis secondary to a higher rate of coronary catheterizations and intracoronary imaging. SCAD is defined as a separation of the coronary artery wall layers not otherwise attributed to atherosclerotic, traumatic, or iatrogenic causes [1]. Although most cases present with a predisposing arterial condition, 20% of cases are found to be idiopathic. Traditional factors found in coronary heart disease, such as atherosclerosis, are not typically seen in these patients. Therefore, younger patients presenting with chest pain should be evaluated for acute coronary syndrome and SCAD should be added to the differential diagnosis. Some factors associated with SCAD include fibromuscular dysplasia, postpartum status, connective tissue disorders, and hormonal therapy [1]. Acute SCAD can be provoked in patients through the introduction of cardio-circulatory stress, such as exercise, emotional stress, recreational drug use, labor, and delivery. Sexual intercourse is a potent cardiovascular stressor, both physically and emotionally. Sexual intercourse should be seen as an exercise, causing changes in blood pressure and heart rate, which can lead to serious complications such as SCAD. Below we discuss a case of SCAD following sexual intercourse and highly recommend physicians to evaluate and educate their high-risk patients regarding aortic dissection with such activities.

## Case presentation

**Figure 1:**
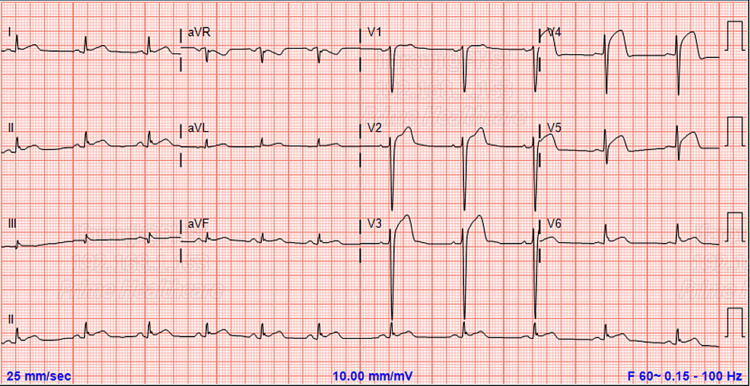
EKG showing significant ST elevation in anterolateral leads.

A 41-year-old African-American gentleman with a history of uncontrolled type 2 diabetes mellitus, and hypertension presented to the emergency room complaining of sudden onset severe chest pain that started immediately after having sexual intercourse with his partner. His symptoms were associated with nausea and sweating. It was the first time he experienced such pain in his life. The pain started one hour prior to the presentation. His home medications were metformin 1,000mg orally twice daily and lisinopril 10mg orally once daily. He has a 10-pack-year smoking history but denied any alcohol or illicit drug abuse. On presentation he was afebrile, heart rate of 74 beats/minute, respiratory rate of 18/minute, and oxygen saturation of 100% on room air. Physical exam was within normal limits, normal S1/S2 on cardiac auscultation without any murmur or extra heart sounds, no jugular venous distension on neck inspection, no crepitation on chest exam, and no lower limb swelling. Initial electrocardiogram (EKG) was significant for ST elevation in anterolateral leads (Figure 1).

**Figure 2:**
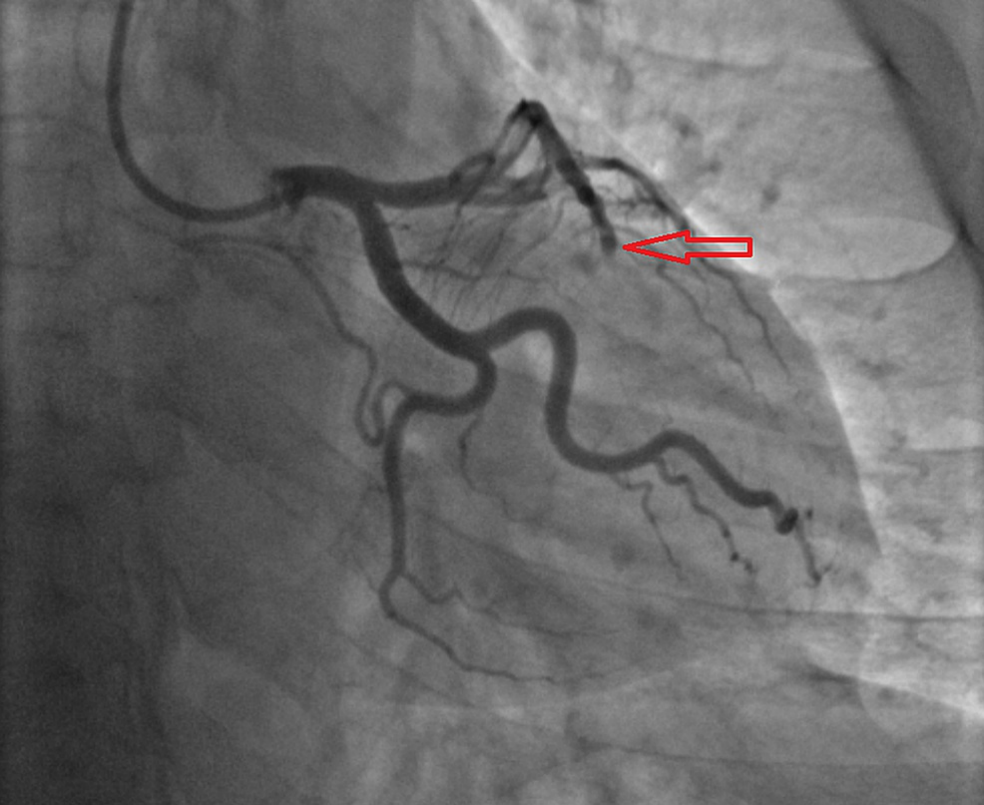
Coronary angiogram was significant for proximal LAD stenosis of 80% likely secondary to local spontaneous dissection and thrombus formation with a 100% thrombus occlusion of the distal LAD (red-arrow). LAD - left anterior descending

He has loaded with aspirin 325mg, ticagrelor 180mg, heparin 5,000 IU IV, and was immediately brought to the catheterization lab. The procedure was done through the right radial artery and the coronary angiogram was significant for proximal left anterior descending (LAD) stenosis of 80% secondary to local spontaneous dissection and thrombus formation with a 100% thrombus occlusion of the distal LAD artery (Figure 2).

A 3.5 x 15mm drug-eluting stent was successfully placed in the proximal LAD, however unsuccessful PCI for the distal LAD with no-reflow phenomenon due to heavy intracoronary clot burden resistant to several attempts of aspiration and local vasodilator injection. After the procedure, the patient was transferred to the cardiac intensive care unit in a stable condition and was continued on tirofiban infusion for 24hrs after the procedure. The patient was started on metoprolol succinate 25mg orally once daily and atorvastatin 80mg orally once daily. His transthoracic echocardiogram showed an ejection fraction of 60% with small apical and apical septal hypokinesis. No evidence of valvular disease was seen on the echocardiogram. Complete blood count and complete metabolic panel were within normal limits. His troponin peak level was 33.8ng/mL. His LDL was 74mg/dL, HDL 44mg/dL, and triglycerides 132mg/dL. The patient was counseled on smoking cessation and discharged home one day after in a stable condition on ticagrelor 90mg orally twice daily and aspirin 81mg orally once daily in addition to metoprolol succinate 25mg orally once daily, lisinopril 10mg orally once daily and atorvastatin 80mg orally once daily. The patient was also started on a basal-bolus insulin regimen, Insulin glargine 10 units at bedtime, and Insulin lispro 10 units subcutaneous three times a day.

## Discussion

SCAD is a unique clinical entity and an important but rare presentation of myocardial infarction (MI). SCAD is defined as a non-iatrogenic, non-traumatic separation of the coronary artery wall and is not associated with atherosclerosis [1]. Although the incidence of reported cases of SCAD has increased over the years secondary to a higher rate of diagnostic coronary artery catheterizations for chest pain evaluation, and the clinical availability and application of high-resolution intracoronary imaging, coronary artery dissection still remains a rare entity [2].

The prevalence of SCAD as a precipitating factor for acute coronary syndrome ranges between 0.1% and 0.4% and is mostly reported in young women without coronary artery disease risk [3,4]. Some factors predisposing a patient to SCAD include pregnancy, postpartum state, and the presence of certain arterial diseases such as fibro-muscular dysplasia, connective tissue disorders autoimmune inflammatory systemic disease [5,6]. Mechanical stressors such as heavy weight lifting, excessive isometric exercise, and emotional stressors can exponentially increase the stress forces in the coronary arterial wall leading to dissection [2,7,8].

In the past, the term SCAD was classified as either atherosclerotic or non-atherosclerotic, but due to the vast differences in pathophysiology and management, it is now more commonly used to define non-atherosclerotic dissections [2,9-11]. These entities should be differentiated as the atherosclerotic dissection is related to plaque erosions or rupture that allows blood flow to reach the intimal space creating tearing force and the non-atherosclerotic is a tear in the intimal layer leading to hematoma formation that in turn causes coronary artery blood flow restrictions [10,12].

Coronary artery dissection can be categorized into type A (ostial dissection), type B (coronary dissection with a false channel), and type C (circumferential detachment with an inner cylinder intussusception) [12]. The most common coronary artery affected is the LAD artery with a rate of up to 70% [13]. Many individuals with SCAD are symptomatic with chest pain being the most reported symptom. Positive troponins are seen in the blood in almost all cases and an initial presentation of fatal arrhythmia or cardiogenic shock has also been reported1 [13].

Sexual intercourse is considered an emotional and physical stressor leading to increased cardiovascular demand. Both blood pressure and heart rate change during different phases of sexual intercourse. Blood pressure and heart rate can have mild-to-moderate changes during sexual activity with a peak at the beginning of pleasure and drop off 10 minutes after orgasm [14]. This activity will maximize the cardiovascular demand promoting and potentially leading to coronary artery dissection in males [15,16]. SCAD triggered by sexual intercourse is extremely rare. To our knowledge, few cases were reported in the literature that is sharing the same presentation of SCAD or even aortic dissection after sexual activity, and the pathophysiology was almost the same [17-19].

The management and treatment of SCAD are extremely challenging due to very limited clinical experiences for this rare entity. Optimal treatment is lacking but many treatment options currently used for patients include conservative management, fibrinolytic therapy, percutaneous coronary intervention (PCI), coronary artery bypass grafting, and cardiac transplantation [20,21].

Revascularization with PCI is recommended in patients presenting with clinical features of MI with ongoing ischemia or hemodynamically instability despite the risks of propagation and extension of the dissection to the side’s branches leading to ongoing ischemia, and worsening hemodynamic instability [1,22]. Medical management is often more suitable for hemodynamically stable patients and includes antiplatelet therapy, beta-blockers, and statin therapy [23]. Beta-blockers may play a fundamental role in the treatment of this disease as it has been shown to decrease future recurrence [24].

## Conclusions

SCAD remains a rare but serious presentation of acute coronary syndrome. Arterial dissection can lead to vascular compromise causing infarction of the myocardium. Patients with SCAD often present with chest pain, but symptoms can range from mild to sudden death. Sexual intercourse has the potential to cause a drastic change in blood pressure and heart rate leading to excessive stress on the vasculature. This stress can lead to tearing and dissection of the arteries. In the case above, we discussed a rare case of post-coital spontaneous coronary artery dissection. Sexual intercourse and how it relates to physiological stress has not been well documented in the literature and how it can affect a person’s health is not readily available to the general population. Hopefully, this case can help increase awareness of SCAD seen with sexual activity and encourage physicians to educate their patients on the topic.

## References

[1] Saw J, Aymong E, Sedlak T (2014). Spontaneous coronary artery dissection: association with predisposing arteriopathies and precipitating stressors and cardiovascular outcomes. Circ Cardiovasc Interv.

[2] Parekh JD, Chauhan S, Porter JL (2020). Coronary Artery Dissection. https://www.ahajournals.org/doi/full/10.1161/CIRCINTERVENTIONS.114.001760.

[3] Daniel EC, Falcão JL (2019). Spontaneous coronary artery dissection - case report and lterature review. Arq Bras Cardiol.

[4] Saw J, Aymong E, Mancini GB, Sedlak T, Starovoytov A, Ricci D (2014). Nonatherosclerotic coronary artery disease in young women. Can J Cardiol.

[5] Basso C, Morgagni GL, Thiene G (1996). Spontaneous coronary artery dissection: a neglected cause of acute myocardial ischaemia and sudden death. Heart.

[6] Vijayaraghavan R, Verma S, Gupta N, Saw J (2014). Pregnancy-related spontaneous coronary artery dissection. Circulation.

[7] Gilhofer TS, Saw J (2019). Spontaneous coronary artery dissection: a review of complications and management strategies. Expert Rev Cardiovasc Ther.

[8] Almaddah NK, Morsy MS, Dishmon D, Khouzam RN (2019). Spontaneous coronary artery dissection: an often unrecognized cause of acute coronary syndrome. Cleve Clin J Med.

[9] Cheung S, Mithani V, Watson RM (2000). Healing of spontaneous coronary dissection in the context of glycoprotein IIB/IIIA inhibitor therapy: a case report. Catheter Cardiovasc Interv Off J Soc Card Angiogr Interv.

[10] Alfonso F (2012). Spontaneous coronary artery dissection: new insights from the tip of the iceberg?. Circulation.

[11] Alfonso F, Bastante T (2014). Spontaneous coronary artery dissection: novel diagnostic insights from large series of patients. Circ Cardiovasc Interv.

[12] Erbel R, Alfonso F, Boileau C (2001). Diagnosis and management of aortic dissection. Eur Heart J.

[13] Tweet MS, Hayes SN, Pitta SR (2012). Clinical features, management, and prognosis of spontaneous coronary artery dissection. Circulation.

[14] Xue-Rui T, Ying L, Da-Zhong Y, Xiao-Jun C (2008). Changes of blood pressure and heart rate during sexual activity in healthy adults. Blood Press Monit.

[15] Gansera L, Deutsch O, Szameitat L, Eichinger W, Gansera B (2016). Aortic dissections type A during sexual intercourse in male patients: accident or systematic coincidence? Examination of 365 patients with acute aortic dissection within 20 years. Thorac Cardiovasc Surg.

[16] Hsu KC, Tsai SH, Kao HW, Chu SJ, Hsu CW (2008). Acute aortic dissection presenting with acute lower-back pain following sexual intercourse. J Intern Med Taiwan.

[17] Schifferdecker B, Pacifico L, Ramsaran EK, Folland ED, Spodick DH, Weiner BH (2004). Spontaneous coronary artery dissection associated with sexual intercourse. Am J Cardiol.

[18] Farouji I, Al-Radideh O, Dacosta T, Shaaban H, Shehadeh A, Suleiman A (2020). Unusual case of spontaneous postcoital type A aortic dissection. Heart Views.

[19] Pierli C, Casini S, del Sordo M, Fineschi M, Bravi A, Leosco D (1995). Post coital coronary artery dissection. Eur Heart J.

[20] Alfonso F, Paulo M, Lennie V (2012). Spontaneous coronary artery dissection: long-term follow-up of a large series of patients prospectively managed with a "conservative" therapeutic strategy. JACC Cardiovasc Interv.

[21] Higgins GL 3rd, Borofsky JS, Irish CB, Cochran TS, Strout TD (2013). Spontaneous peripartum coronary artery dissection presentation and outcome. J Am Board Fam Med.

[22] Hayes SN, Kim ES, Saw J (2018). Spontaneous coronary artery dissection: current state of the science: a scientific statement from the American Heart Association. Circulation.

[23] Saw J, Ricci D, Starovoytov A, Fox R, Buller CE (2013). Spontaneous coronary artery dissection: prevalence of predisposing conditions including fibromuscular dysplasia in a tertiary center cohort. JACC Cardiovasc Interv.

[24] Saw J, Humphries K, Aymong E, Sedlak T, Prakash R, Starovoytov A, Mancini GB (2017). Spontaneous coronary artery dissection: clinical outcomes and risk of recurrence. J Am Coll Cardiol.

